# Splenic artery embolization changes the management of blunt splenic injury: an observational analysis of 680 patients graded by the revised 2018 AAST-OIS

**DOI:** 10.1007/s00464-022-09531-0

**Published:** 2022-08-12

**Authors:** Being-Chuan Lin, Cheng-Hsien Wu, Yon-Cheong Wong, Huan-Wu Chen, Chen-Ju Fu, Chen-Chih Huang, Chen-Te Wu, Chi-Hsun Hsieh

**Affiliations:** 1 Division of Trauma and Emergency Surgery, Department of Surgery, Chang Gung Memorial Hospital, Chang Gung University, 5, Fu-Hsing Street, Kwei-Shan, Tao-Yuan City, 333 Taiwan; 2 Division of Emergency and Critical Care Radiology, Department of Medical Imaging and Intervention, Chang Gung Memorial Hospital, Chang Gung University, Tao-Yuan City, Taiwan; 3 Department of Medical Imaging and Intervention, New Taipei Municipal Tucheng Hospital, Chang Gung Medical Foundation, New Taipei City, Taiwan

**Keywords:** Splenic artery embolization, Blunt splenic injury, Splenic salvage rate, Pseudoaneurysm, Contrast extravasation

## Abstract

**Background:**

This study aimed to evaluate the management of blunt splenic injury (BSI) and highlight the role of splenic artery embolization (SAE).

**Methods:**

We conducted a retrospective review of all patients with BSI over 15 years. Splenic injuries were graded by the 2018 revision of the American Association for the Surgery of Trauma-Organ Injury Scale (AAST-OIS). Our hospital provide 24/7 in-house surgeries and 24/7 in-house interventional radiology facility. Patients with BSI who arrived hypotensive and were refractory to resuscitation required surgery and patients with vascular injury on abdominal computed tomography were considered for SAE.

**Results:**

In total, 680 patients with BSI, the number of patients who underwent nonoperative management with observation (NOM-obs), SAE, and surgery was 294, 234, and 152, respectively. The number of SAEs increased from 4 (8.3%) in 2001 to 23 (60.5%) in 2015 (*p* < 0.0001); conversely, the number of surgeries decreased from 21 (43.8%) in 2001 to 4 (10.5%) in 2015 (*p* = 0.001). The spleen-related mortality rate of NOM-obs, SAEs, and surgery was 0%, 0.4%, and 7.2%, respectively. In the SAE subgroup, according to the 2018 AAST-OIS, 234 patients were classified as grade II, *n* = 3; III, *n* = 21; IV, *n* = 111; and V, *n* = 99, respectively.; and compared with 1994 AST-OIS, 150 patients received a higher grade and the total number of grade IV and V injuries ranged from 96 (41.0%) to 210 (89.7%) (*p* < 0.0001). On angiography, 202 patients who demonstrated vascular injury and 187 achieved hemostasis after SAE with a 92.6% success rate. Six of the 15 patients failed to SAE preserved the spleen after second embolization with a 95.5% salvage rate.

**Conclusions:**

Our data confirm the superiority of the 2018 AAST-OIS and support the role of SAE in changing the trend of management of BSI.

**Graphical abstract:**

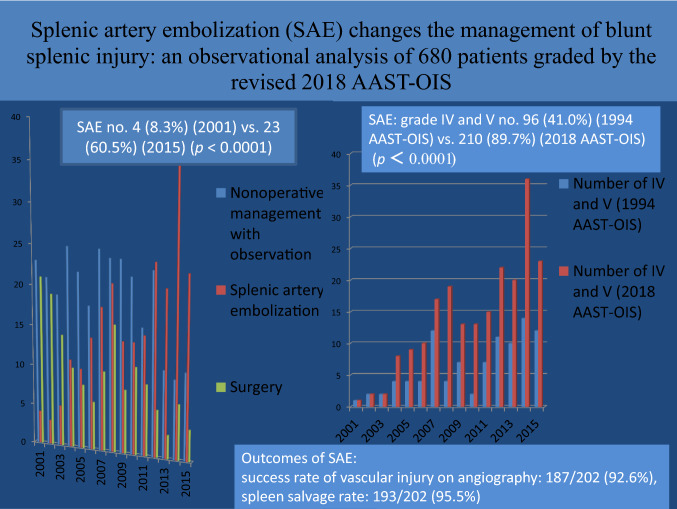

Over the last 30 years, there has been a prominent shift towards a more conservative approach to the management of blunt splenic injury (BSI), with an emphasis on nonoperative management (NOM) [1–4]. NOM ranges from observation alone (NOM-obs) to splenic artery embolization (SAE) with the aim of preserving the spleen and its function. In 1997, Clancy et al. reported that surgery remained the most common treatment in 1059 patients with BSI, being performed in 58.3% (splenorrhaphy, 11.3%; splenectomy, 47.0%) of patients, and NOM-obs in 41.7% [5]. However, contemporaneously, Sclafani et al. prospectively collected 172 patients with BSI; 61 (35.5%) underwent SAE, with a 95.1% spleen salvage rate and 1.7% spleen-related mortality rate [6]. The potential benefits of SAE include avoidance of nontherapeutic laparotomies, lower rates of intra-abdominal complications and blood transfusions, maintenance of immunological function, and a shorter hospital course. Currently, with the improvement of contrast-enhanced multidetector computed tomography (MDCT) and application of SAE, NOM has become the standard of care for BSI and higher NOM rates of up to 80–90%, with success in 90% of cases, have been reported [7–11]. This study aimed to evaluate the management of patients with BSI and highlight the evolving role of SAE. We incorporated vascular injury and graded our patients and analyzed the outcomes.

## Materials and methods

### Study design and period

The study covered a 15-year period from 1 January 2001 to 31 December 2015. The trauma registry and medical records of patients with BSI at our level 1 trauma center were reviewed retrospectively. This study was approved by the Institutional Review Board of Chang Gung Memorial Hospital (reference number: 201900640B0). The need for informed consent was waived, as the data were collected from existing patient records, and the de-identification standard was followed to protect the confidentiality of personal information. The STROBE guidelines were observed [12].

### Study population

Our hospital in Northern Taiwan is a 3,704-bed level 1 trauma center with a well-established team that includes 24/7 in-house year-round attending trauma surgeons and 24/7 in-house attending interventional radiologists. The operating room and angiographic suite are available 24 h a day. Patients with an Abbreviated Injury Score code indicating splenic injury were included. Patients were excluded if they died in the emergency department or had penetrating injuries.

### Injury grading and resuscitation

All patients had splenic injuries documented on their admission abdominal CT scans or surgical findings; these scans were graded and interpreted by trauma surgeons and critical care radiologists for evidence of vascular injury (contrast extravasation, pseudoaneurysm, arteriovenous fistula, or vessel truncation). All the splenic injuries were graded according to the 2018 revision of the American Association for the Surgery of Trauma-Organ Injury Scale (AAST-OIS), which incorporates vascular injury on abdominal CT (Table 1) [13]. Patients admitted to this institution were resuscitated according to the standard Advanced Trauma Life Support protocol for major trauma.

**Table 1:** Spleen Organ Injury Scale—2018 Revision

AAST Grade	AIS Severity	Imaging criteria (computed tomography findings)
I	2	Subcapsular hematoma < 10% surface area
		Parenchymal laceration < 1 cm depth
		Capsular tear
II	2	Subcapsular hematoma 10–50% surface area; intraparenchymal hematoma < 5 cm
		Parenchymal laceration 1–3 cm
III	3	Subcapsular hematoma > 50% surface area; ruptured subcapsular or intraparenchymal hematoma ≥ 5 cm
		Parenchymal laceration > 3 cm depth
IV	4	Any injury in the presence of a splenic vascular injury or active bleeding confined within splenic capsule
		Parenchymal laceration involving segmental or hilar vessels producing > 25% devascularization
V	5	Any injury in the presence of splenic vascular injury with active bleeding extending beyond the spleen into the peritoneum
		Shattered spleen

Vascular injury is defined as a pseudoaneurysm or arteriovenous fistula and appears as a focal collection of vascular contrast that decreases in attenuation with delayed imaging. Active bleeding from a vascular injury presents as vascular contrast, focal or diffuse, that increases in size or attenuation in delayed phase. Vascular thrombosis can lead to organ infarction. Grade based on highest grade assessment made on imaging, at operation or on pathologic specimen.More than one grade of splenic injury may be present and should be classified by the higher grade of injury. Advance one grade for multiple injuries up to a grade III. *AAST* American association for the surgery of trauma, *AIS* abbreviated injury scale

### Management of protocol

**Fig. 1:**
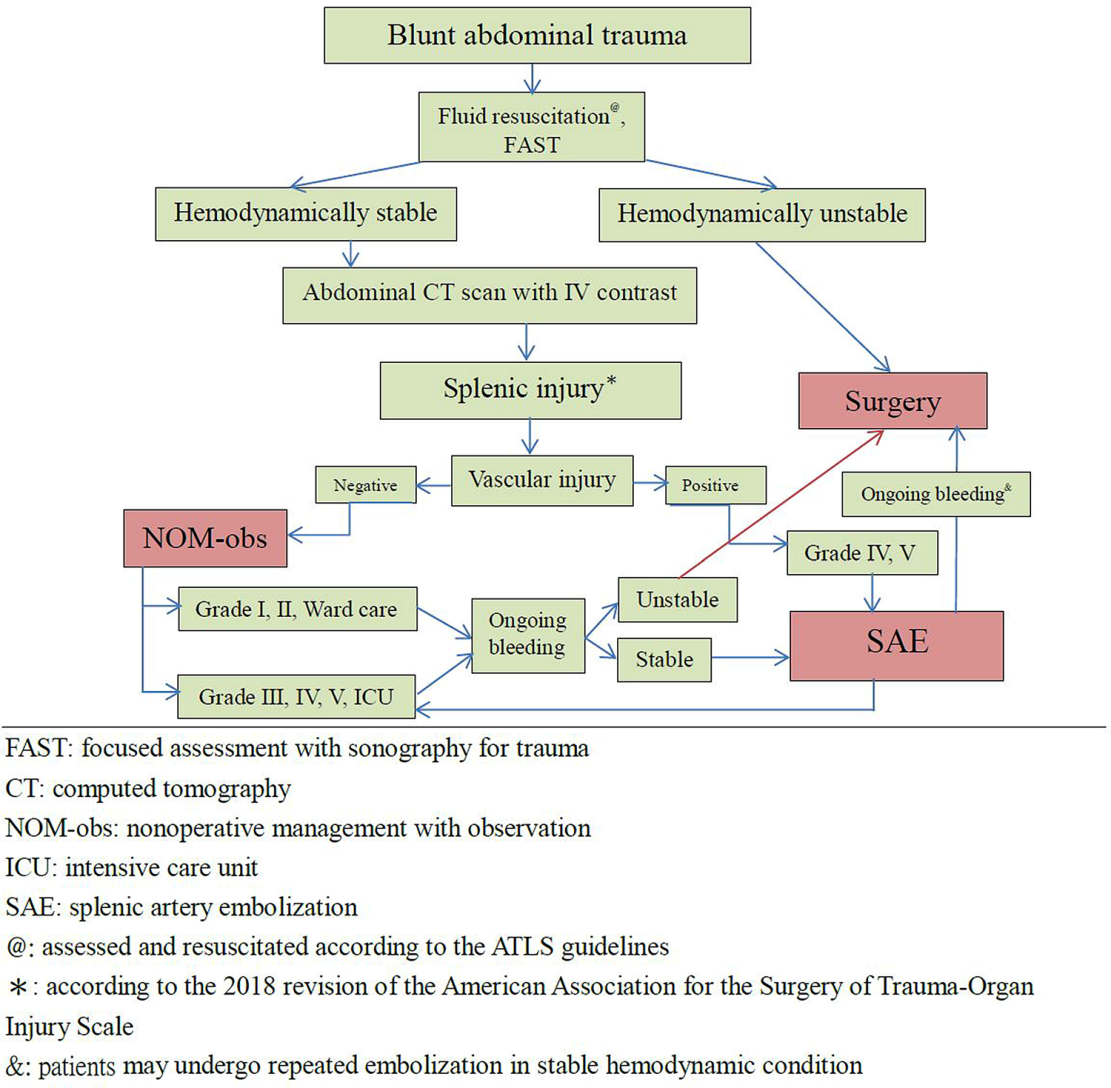
Algorithm for management of patients with blunt splenic injury

Patients with BSI who arrive hypotensive and are refractory to resuscitation require surgery. In our institution, all hemodynamically stable patients (including those with shock at triage and response to resuscitation) with grade I or II splenic injuries on admission abdominal CT are observed in the ward. Patients with grades III, IV, or V without vascular injury on abdominal CT undergo NOM-obs in the intensive care unit (ICU). Patients with vascular injury, regardless of grade, on abdominal CT are considered for SAE (Fig. 1). Patients with a continuous decrease in hemoglobin levels with ongoing transfusion requirements in the course of attempting NOM-obs are also considered for SAE. SAE is not appropriate for generalized peritonitis or for patients with other intra-abdominal injuries requiring surgery. After SAE, these patients are managed in the ICU for 2 days with close monitoring of their hemodynamic parameters, intra-abdominal pressure, and hemoglobin, as well as serial abdominal examinations.

### Technique of SAE

Catheterization was performed using the Seldinger method through the right or left common femoral artery. Celiac angiography was performed to delineate the splenic artery and side branches, including the dorsal and great pancreatic arteries, and to identify active contrast extravasation, pseudoaneurysm, degree of devitalized spleen, and abnormally truncated vessels. Proximal embolization is defined as the introduction of the embolization material into the splenic artery trunk. For distal embolization, the microcatheter is placed beyond the splenic hilum and embolize a segmental branch distal to any major potential collateral pathways to preserve as much of the spleen as possible. Combined embolization is defined by the combination of both techniques. Various agents, such as microcoils or Gelfoam cubes (1–3 mm in size) (Upjohn, Inc., Kalamazoo, MI) can be used either alone or in combination. The SAE procedures were discontinued when cessation of contrast medium extravasation and arterial occlusion were achieved.

### Definition

NOM-obs was defined as conservative management with close observation or serial laboratory data follow-up of hemoglobin q8h for 2 days. Failure of NOM-obs was defined as further splenic bleeding necessitating subsequent SAE or splenic surgery. Pseudoaneurysm was defined as contrast medium confined within the splenic capsule. Contrast extravasation was defined as free spillage of contrast medium extending beyond the spleen into the peritoneum. Success of the SAE was defined as achieving hemostasis after the first embolization. Failure of the SAE was defined as failure to achieve hemostasis and required additional intervention (repeated SAE or splenic surgery). Spleen salvage was defined as patient discharge with the spleen in situ. The overall mortality was defined as in-hospital death from any cause. Spleen-related mortality was defined as death directly due to acute ongoing bleeding from uncontrolled BSI. Complications were graded as minor or major according to the Clavien-Dindo classification (CDC) [14]. Grade I and II complications did not require treatment or only medical treatment and were classified as minor complications. Grade III, IV, and V complications required endoscopic or surgical treatment were life-threatening or resulted in death, and were classified as major complications [14].

### Demographic data analysis

The medical charts were reviewed retrospectively with respect to age, sex, trauma mechanism, injury severity score (ISS), imaging study, type of management, and outcomes.

### Statistics

Categorical data are presented as numerical values and continuous data as median (i.q.r.) values. Fisher’s exact test or Pearson’s *χ*^*2*^ test was used for comparisons of categorical data, as appropriate. The Mann–Whitney *U* test was used for continuous data. All statistical analyses were performed using SPSS® version 20.0 (IBM, Armonk, New York, USA). A *p *value < 0.05 (two‐sided) was considered to indicate statistical significance.

## Results

### Characteristics of the study population and management

**Fig. 2:**
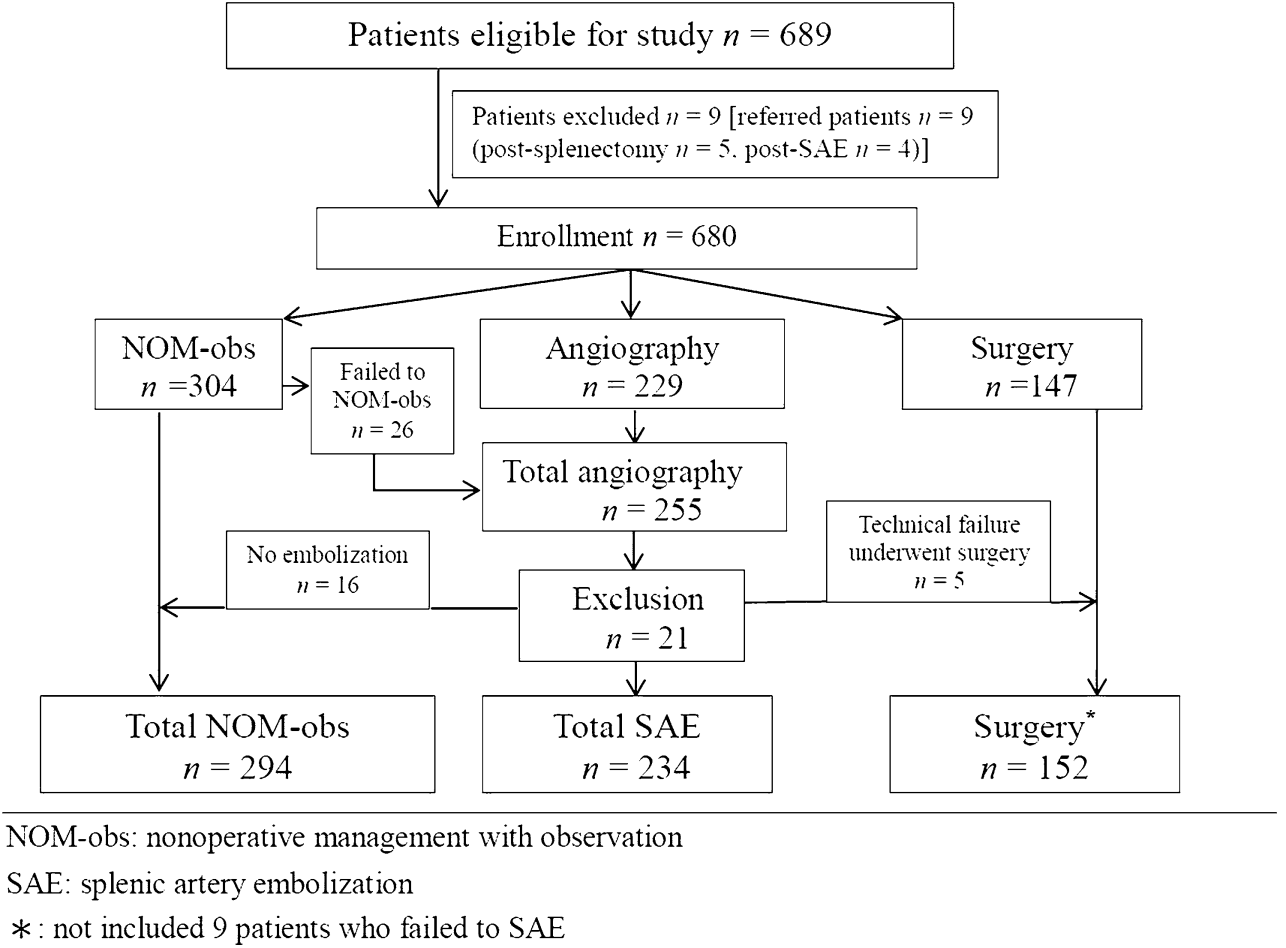
Flow diagram of patients with blunt splenic injury in Chang Gung Memorial Hospital from 2001 to 2015

**Fig. 3:**
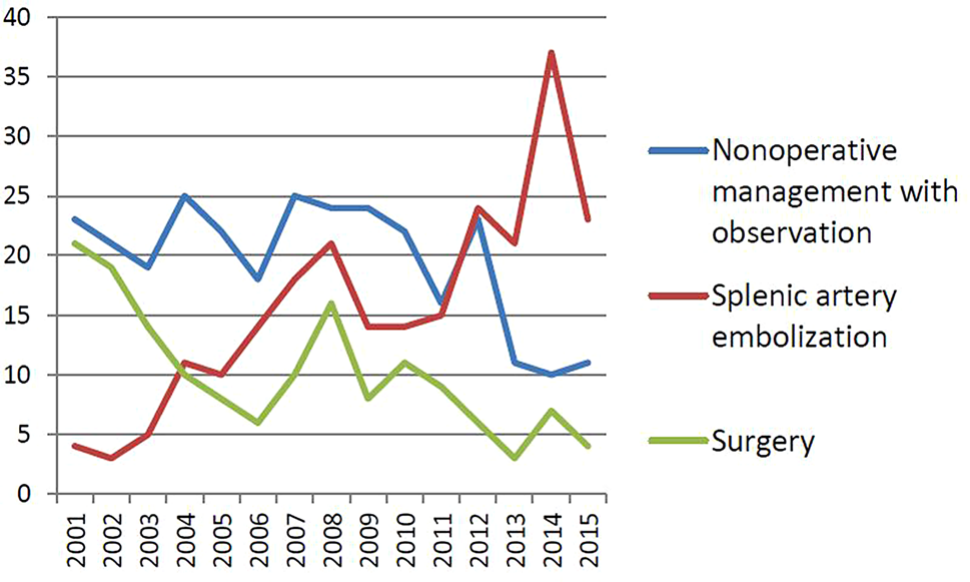
Patients with blunt splenic injury in Chang Gung Memorial Hospital (2001–2015, *n* = 680)

**Fig. 4:**
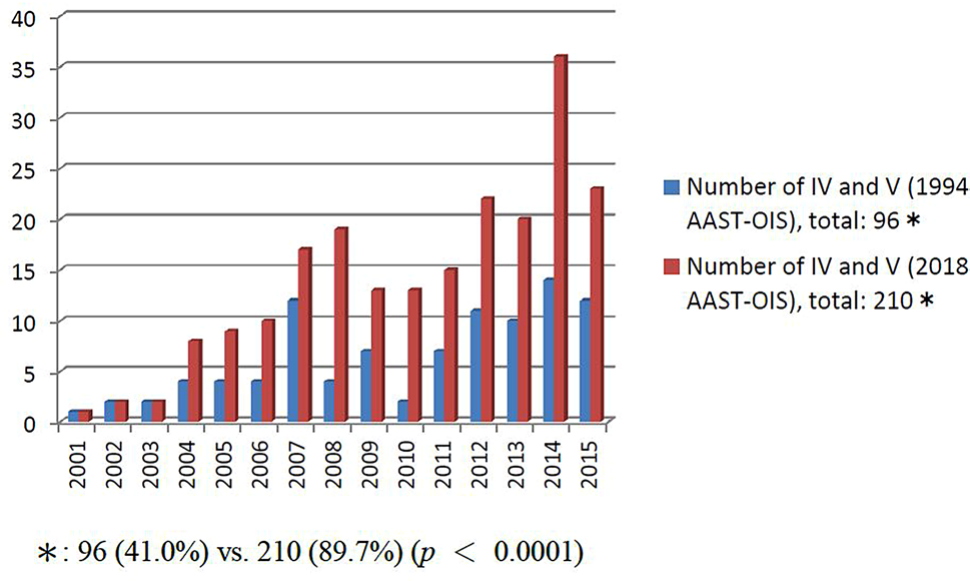
Compared with 1994 AAST-OIS, the total number of grade IV and V injuries graded by 2018 AAST-OIS ranged from 96 (41.0%) to 210 (89.7%) (*p* < 0.0001)

Our trauma care is mainly based on the blunt injury (blunt vs penetrating abdominal injury admission, 92 vs 8%). Of the study period, five patients were penetrating splenic injury, and they were excluded from this study. In total, 689 patients with BSI were managed during this period; the nine of these who were treated at another facility (splenectomy, *n* = 5; SAE, *n* = 4) were excluded. Of the remaining 680 patients, 294 (43.2%), 234 (34.4%), and 152 (22.4%) eventually underwent NOM-obs, SAE, and surgery, respectively (Fig. 2). The trend of change in the management is presented in Fig. 3. The demographic data and clinical characteristics of the NOM-obs, SAE, and surgery groups are presented in Table 2. According to 1994 AAST-OIS, 294 patients managed with NOM-obs were classified as grade I, *n* = 23; II, *n* = 109; III, *n* = 125; IV, *n* = 35; and V, *n* = 2, respectively. With incorporation of vascular injury and graded by 2018 AAST-OIS, 294 patients were classified as grade I, *n* = 23; II, *n* = 108; III, *n* = 125; IV, *n* = 34; and V, *n* = 4, respectively (Table 2). Only two patients (grade II, *n* = 1; IV, *n* = 1) were graded higher to grade V. In the SAE subgroup, according to the 1994 AAST-OIS, 234 patients were classified as grade I, *n* = 1; II, *n* = 22; III, *n* = 115; IV, *n* = 85; and V, *n* = 11, respectively. However, with the incorporation of vascular injury, 150 patients received a higher grade, including 62 patients (grade II, *n* = 12; III, *n* = 50) who were classified as 2018 AAST-OIS grade IV, and 88 patients (grade I, *n* = 1; II, *n* = 7; III, *n* = 44; IV, *n* = 36) as 2018 AAST-OIS grade V. Consequently, graded by 2018 AAST-OIS, 234 patients were classified as grade I, *n* = 0; II, *n* = 3; III, *n* = 21; IV, *n* = 111; and V, *n* = 99 (Table 2), respectively; and the total number of grade IV and V injuries ranged from 96 (41.0%) (1994 AAST-OIS) to 210 (89.7%) (2018 AAST-OIS) (*p* < 0.0001) (Fig. 4). In the surgery subgroup, there was no difference since the severity was graded by operative findings and not by images. Our data demonstrated a significant difference between the 1994 AAST and 2018 AAST-OIS, particularly, in the SAE subgroup. According to the 2018 AAST-OIS, the median ISS for NOM-obs, SAE, and surgery was 9, 25, and 25, respectively. In the NOM-obs subgroup, one patient died of associated head injury, and no deaths were spleen-related. In the SAE subgroup, four patients died of associated head injury, and one died of splenic injury. In the surgery subgroup, 14 patients died of associated injuries (head, *n* = 5; liver, *n* = 5; lung, *n* = 2, superior mesenteric artery, *n* = 1; pelvis, *n* = 1), and 11 patients died of splenic injury. The spleen-related mortality rate of NOM-obs, SAEs, and surgery was 0, 0.4, and 7.2%, respectively (Table 2). The median length of hospital stay (days) for NOM-obs, SAE, and surgery was 6, 10, and 12, respectively (Table 2).

**Table 2:** Demographic data and clinical characteristics of 680 patients with BSI who underwent NOM-obs, SAE, and surgery

Characteristics	NOM-obs	SAE	Surgery
Total patients	680		
No. of patients (%)	294 (43.2)	234 (34.4)	152 (22.4)
Sex			
Male, *n* (%)	199 (67.7)	189 (80.8)	121 (79.6)
Female, *n* (%)	95 (32.3)	45 (19.2)	31 (20.4)
Age (years)*	25 (18–44)	34 (23–50.5)	30 (21–48)
Shock at triage, *n* (%)	9 (3.1)	43 (18.4)	105 (69.1)
2018 AAST-OIS			
I, *n* (%)	23 (7.8)	0	6 (3.9)
II, *n* (%)	108 (36.7)	3 (1.3)	22 (14.5)
III, *n* (%)	125 (42.5)	21 (9.0)	32 (21.0)
IV, *n* (%)	34 (11.6)	111 (47.4)	34 (22.4)
V, *n* (%)	4 (1.4)	99 (42.3)	58 (38.2)
OIS*	3 (2–3)	4 (4–5)	4 (3–5)
Injury severity score*	9 (5–14.5)	25 (18–29)	25 (17–34)
Length of stay (days)*	6 (5–8)	10 (7–15)	12 (7–17)
Spleen-related morbidity^a^, *n* (%)	1 (0.3)	22 (9.4)	9 (5.9)
Splenic abscess, *n* (%)	1 (0.3)	7 (3.0)	1 (0.6)
Rebleeding underwent surgery, *n* (%)	0	9 (3.8)	**7**^**b**^ (4.6)
Underwent SAE, *n* (%)	0	6 (2.6%)	1 (0.6%)
Overall mortality, *n* (%)	1 (0.3)	5 (2.1)	25 (16.4)
Spleen-related mortality, *n* (%)	0	1 (0.4)	11 (7.2)

*BSI* blunt splenic injury, *NOM-obs* nonoperative management with observation, *SAE* splenic artery embolization, 2018 *AAST-OIS* 2018 revision of the American Association for the Surgery of Trauma-Organ Injury Scale

*Values are median (i.q.r.), ^a^complication required intervention, ^b^2nd surgery

### SAE and outcomes

Of the 234 patients who underwent SAE, 189 were male and 45 were female, and the median age of each gender was 34.0 years. The median initial systolic blood pressure (mmHg) was 116.0 (Table 3). Forty-three patients (18.4%) presented with shock at triage and underwent SAE after resuscitation, and the demographic data and outcomes are presented in Table 4. The most frequent cause of injury was motorcycle accidents (*n* = 151, 64.5%), followed by falling (*n* = 35, 15.0%) (Table 3). Laboratory data revealed a median initial serum hemoglobin level (g/dL) of 11.4. The total number of patients with grade IV (*n* = 111, 47.4%) and V (*n* = 99, 42.3%) injuries accounted for 89.7% of injuries. The median ISS was 25.0 and the percentage of multiple injuries was 67.1% (Table 3). The three most common indications for SAE were (1) pseudoaneurysm (*n* = 91, 38.9%), (2) contrast extravasation (*n* = 53, 22.6%), and (3) combined contrast extravasation with pseudoaneurysm (*n* = 43, 18.4%) on abdominal CT (Table 3). The median time from injury to SAE was 7 h, and nine patients underwent concomitant embolization for associated injuries (kidney, *n* = 5; liver, *n* = 3; pelvis, *n* = 1). On angiography, 32 patients (13.7%) demonstrated no evidence of vascular injury on angiography and were embolized empirically (Table 3). To reveal the effect of embolization hemostasis on splenic vascular injury, we focused on 202 patients (86.3%) who demonstrated vascular injury (pseudoaneurysm, *n* = 128; combined contrast extravasation with pseudoaneurysm, *n* = 36; and contrast extravasation, *n* = 35) on angiography. Of these 202 patients, the number of patients who underwent proximal, distal, and combined embolization was 64 (31.7%), 84 (41.6%), and 54 (26.7%), respectively, microcoils alone were the most common choice of embolizer (*n* = 166, 82.2%) and 187 patient achieved hemostasis after embolization with a 92.6% success rate (Table 5). Of the 15 patients who failed to SAE with ongoing bleeding, spleen salvage after a second SAE was possible in 6, with a 95.5% salvage rate (Table 5); however, the other nine patients underwent splenectomy. Outcomes of the various grades of 202 patients with vascular injury on angiography who underwent SAE are presented in Table 6. Of the 202 patients underwent SAE, six patients developed splenic abscesses and required further intervention (CT-guided drainage, *n* = 5; surgery, *n* = 1) (Table 5). One patient died of splenic rebleeding despite underwent emergent surgery with a spleen-related mortality rate of 0.4% (Table 2).

**Table 3:** Demographic data and clinical characteristics of 234 patients with BSI who underwent SAE

Sex	
Male, *n* (%)	189 (80.8)
Female, *n* (%)	45 (19.2)
Age (years)*	34 (23–50.5)
Transferred case, *n* (%)	147 (62.8)
Shock at triage, *n* (%)	43 (18.4)
SBP*	116 (96–137)
HR*	96 (82–112)
RR*	20 (18–20)
Mechanism	
Motorcycle, *n* (%)	151 (64.5)
Fall, *n* (%)	35 (15.0)
Motorcar, *n,* (%)	19 (8.1)
Assault, *n* (%)	12 (5.1)
Others (bicycle, compression, passenger etc.), *n* (%)	17 (7.3)
Initial serum hemoglobin (g/dL)*	11.4 (9.4–13.4)
Grade of splenic injury	
2018 AAST-OIS	
II, *n* (%)	3 (1.3)
III, *n* (%)	21 (9.0)
IV, *n* (%)	111 (47.4)
V, *n* (%)	99 (42.3)
OIS*	4 (4–5)
Isolated splenic injury, *n* (%)	77 (32.9)
Multiple injuries, *n* (%)	157 (67.1)
Injury severity score*	25 (18–29)
Indication of SAE	
Abdominal CT, *n* (%)	208 (88.9)
Pseudoaneurysm, *n* (%)	91 (38.9
Contrast extravasation, *n* (%)	53 (22.6)
Combined contrast extravasation with pseudoaneurysm, *n* (%)	43 (18.4)
High injury grade, *n* (%)	18 (7.7)
Large hemoperitoneum, *n* (%)	3 (1.3)
Failed to NOM-obs, *n* (%)	26 (11.1)
Time from injury to SAE (hours)*	7 (5–27.3)
Vascular injury on angiography, *n* (%)	
Positive, *n* (%)	202 (86.3)
Negative, *n* (%)	32 (13.7)

*BSI* blunt splenic injury, *SAE* splenic artery embolization, 2018 *AAST-OIS* 2018 revision of the American Association for the Surgery of Trauma-Organ Injury Scale, *CT* computed tomography, *NOM-obs* nonoperative management with observation

*Values are median (i.q.r.)

**Table 4:** Comparison of demographic data and clinical characteristics of 234 patients with BSI who presented with shock and nonshock at triage and underwent SAE

Characteristics	Shock	Nonshock	*p* value
No. of patients	43	191	
Sex			
Male, *n* (%)	35 (81.4)	154 (80.6)	> 0.999
Female, *n* (%)	8 (18.6)	37 (19.4)	
Age (years)*	40 (20–50)	34 (23–52)	0.855
Initial serum hemoglobin (g/dL)*	10.7 (8.9–13.3)	11.5 (9.4–13.5)	0.168
2018 AAST-OIS			
II, *n* (%)	1 (2.3)	2 (1.0)	0.268
III, *n* (%)	3 (7.0)	18 (9.4)	
IV, *n* (%)	16 (37.2)	95 (49.7)	
V, *n* (%)	23 (53.5)	76 (39.8)	
ISS*	25 (19–33)	25 (18–29)	0.146
Time from injury to SAE (hours)*	6 (4.5–17)	7 (5–28)	0.204
Angiographic findings:			0.356
Vascular injury, *n* (%)	39 (90.7)	163 (85.3)	
No vascular injury, *n* (%)	4 (9.3)	28 (14.7)	
Success of SAE^#^, *n* (%)	37 (94.9)	150 (92.0)	> 0.999
Re-embolization^#^, *n* (%)	1 (2.6)	5 (3.1)	> 0.999
Surgery^#^, *n* (%)	1 (2.6)	8 (4.9)	> 0.999
Length of stay (days)*	10 (7–15)	9 (7–14)	0.502

*BSI* blunt splenic injury, *SAE* splenic artery embolization, 2018 *AAST-OIS* 2018 revision of the American Association for the Surgery of Trauma-Organ Injury Scale, *ISS* injury severity score

*Values are median (i.q.r.), ^#^specific to patients with vascular injury on angiography

**Table 5:** Outcomes of 202 patients with BSI who demonstrated vascular injury on angiography and underwent SAE

Angiographic findings	
Pseudoaneurysm, *n* (%)	128 (63.4)
Combined contrast extravasation with pseudoaneurysm, *n* (%)	36 (17.8)
Contrast extravasation, *n* (%)	35 (17.3)
Arteriovenous fistula, *n* (%)	3 (1.5)
Embolization method	
Proximal, *n* (%)	64 (31.7)
Distal, *n* (%)	84 (41.6)
Combined, *n* (%)	54 (26.7)
Success of SAE, *n* (%)	187 (92.6)
Failure of SAE, *n* (%)	15 (7.4)
Splenectomy, *n* (%)	8 (3.9)
Repeated SAE → splenectomy, *n* (%)	1 (0.5)
Repeated SAE, *n* (%)	6 (3.0)
Spleen salvage, *n* (%)	193 (95.5)
Post-SAE splenic abscess underwent intervention, *n* (%)	6 (3.0)
CT-guided drainage, *n* (%)	5 (2.5)
Surgery, *n* (%)	1 (0.5)

*BSI* blunt splenic injury, *SAE* splenic artery embolization, *CT* computed tomography

**Table 6:** Outcomes of the various grades of 202 patients with BSI who demonstrated vascular injury on angiography and underwent SAE

2018 AAST-OIS, *n*	Outcomes	
	Success of SAE,* n* (%)	Spleen salvage, *n* (%)
II, 3	3 (100)	3 (100.0)
III, 14	13 (92.9)	13 (92.9)
IV, 95	89 (94.6)	92 (96.8)
V, 90	82 (91.9)	85 (94.4)
Total, 202	187 (92.6)	193 (95.5)

*BSI* blunt splenic injury, 2018 *AAST-OIS* 2018 revision of the American Association for the Surgery of Trauma-Organ Injury Scale, *SAE* splenic artery embolization

## Discussion

The management of patients with BSI continues to evolve. The application of NOM in hemodynamically stable patients with BSI is widely accepted and has become standard in recent decades [2–4]. Previously, hemodynamically unstable patients with BSI were managed surgically. However, with the evolution of damage control resuscitation and implementation of the permissive hypotension strategy, interventional radiology has extended the application of NOM, especially using SAE in patients with BSI [15, 16]. Shock is not an absolute contraindication for SAE [15]. In our series, 39 of 43 patients who presented with shock at triage and demonstrated vascular injury on angiography underwent SAE with a 94.9% success rate, with no statistically significant differences when compared with the nonshock group (94.9% vs 92.0%, *p* > 0.999) (Table 4). Since 1998, we have performed transarterial embolization as an adjunctive procedure to NOM in selected patients with blunt abdominal trauma [17]. Of the 680 patients with BSI over a 15-year period, 294 (43.2%) underwent NOM-obs, 234 (34.4%), SAE, and 152 (22.4%), surgery. The severity of injury and proportion of high-grade injuries in these three subgroups were analyzed. Of this study, the median ISS/total number of grade IV and V injuries was 9 / 37 (12.6%), 25 / 210 (89.7%), and 25/92 (60.6%) in NOM-obs, SAE, and surgery, respectively. This could reflect the complexity of multisystem, major injuries in our patients, especially in the SAE and surgery subgroups. During the study period, an algorithm for management of BSI was established and updated to reflect the increasing prevalence of contrast-enhanced MDCT (Fig. 1). The benefits of SAE include its feasibility and efficacy; as these became more apparent, the number of SAEs steadily and significantly increased from 4 (8.3%) in 2001 to 23 (60.5%) in 2015 (*p* < 0.0001), and the number of patients who underwent surgery declined from 21 (43.8%) in 2001 to 4 (10.5%) in 2015 (*p* = 0.001) (Fig. 3). To our knowledge, this is the largest series of SAEs in BSI from a single trauma center. A review of the literature demonstrates that a higher failure rate of NOM-obs in BSI is associated with an increasing grade of injury, especially in patients with grade IV and V injury; the failure rate exceeded 50% [18, 19]. The use of SAE has been recommended in high-grade injuries to reduce the failure rate to 4–10% [2–4, 10, 20, 21] and SAE has been increasingly advocated for as an important adjunct to NOM [2–5, 7–11, 22–25]. Apart from in high-grade injury, the presence of vascular injury, such as contrast extravasation or pseudoaneurysms around or within the spleen demonstrated on abdominal CT, has been strongly associated with the failure of NOM-obs [26]. With the increasing use of contrast-enhanced MDCT, better characterization of splenic vascular injury is possible, which can alert the clinician to the severity of injury and facilitate early SAE or surgery for BSI. However, the 1994 AAST-OIS did not include vascular injury. In 2007, Marmery et al. proposed a new system (Baltimore CT Severity Index) that incorporated vascular injury for splenic injuries [27]. This was superior to the 1994 AAST-OIS in predicting the need for embolization or surgery [28]. With the incorporation of vascular injury, the risk of ongoing bleeding can be prevented and the injury assigned a lower grade despite the presence of contrast extravasation or pseudoaneurysm on imaging. The AAST accordingly published a 2018 update for their classification of splenic injuries that included the imaging features of contrast extravasation and pseudoaneurysm to improve the system’s value for clinical patient management [13]. According to the 2018 AAST-OIS, any injury in the presence of a splenic vascular injury (pseudoaneurysm or arteriovenous fistula) or active bleeding confined within the splenic capsule is grade IV, and active bleeding extending beyond the spleen into the peritoneum is grade V (Table 1) [13]. Unlike most of the published reports in which patients were graded according to the 1994 AAST-OIS, we incorporated vascular injury and graded our patients using the 2018 AAST-OIS. We reviewed abdominal CTs and found that 187 patients (79.9%) in the SAE subgroup had a vascular injury; pseudoaneurysm was the most common finding (*n* = 91), followed by contrast extravasation (*n* = 53) and combined contrast extravasation with pseudoaneurysm (*n* = 43) (Table 3). According to the 1994 AAST-OIS, 234 patients who underwent SAE were classified as grade I, *n* = 1; II, *n* = 22; III, *n* = 115; IV, *n* = 85; and V, *n* = 11, respectively. However, with the incorporation of vascular injury, 234 patients were classified as grade I, *n* = 0; II, *n* = 3; III, *n* = 21; IV, *n* = 111; and V, *n* = 99 (Table 2), respectively; the difference between the 1994 AAST-OIS and 2018 AAST-OIS in the total number of grade IV and V injuries was statistically significant (*p* < 0.0001) (Fig. 4). On angiography, 202 patients demonstrated vascular injury with an 86.3% positive rate *(*Table 3), and the vascular injury rate of grades IV and V was 88.1%; however, grades II and III only had a 70.8% vascular injury rate (*p* = 0.029). The high proportion of grades IV and V (*n* = 210, 89.7%) with a high vascular injury rate on angiography (*n* = 185, 88.1%) reflected the severity of injury accurately and highlighted the strictness of the classification of high-grade injury for SAE in our series. Thirty-two patients had no evidence of vascular injury on the initial angiogram and embolized empirically. In patients without vascular injury on angiography, the need for embolization was less clear. It has been reported that approximately 10% of patients with a negative splenic angiogram in the setting of trauma will require further angiographic evaluation or a subsequent operation [28]. Subsequent rebleeding may occur because some vascular injuries are initially not detected on CT or angiography because of vasospasm [29]. In a 2011 meta-analysis by Schnuriger et al. that included 15 retrospective studies with 479 patients, the overall failure rate of SAE was 10.2%, with rebleeding being the most common cause [8]. Of the 202 patients who demonstrated vascular injury on angiography, 187 achieved hemostasis after embolization with a 92.6% success rate. The remaining 15 patients (grade III, *n* = 1; IV, *n* = 6; V, *n* = 8) failed to undergo SAE and nine of these underwent splenectomy; splenic preservation was possible in the other 6 after a second SAE, with a 95.5% spleen salvage rate (Table 5), which is in accordance with previous reports [5, 7, 8, 11, 30–32]. We analyzed the reason for failure: recurrent pseudoaneurysm on repeated angiography in six patients, persistent extravasation on angiography in two, and coil migration in one. Recurrent pseudoaneurysms were suspected in the six patients who underwent surgery**.** SAEs can be performed in a proximal, distal, or combined manner [8, 11, 33]. Of the 202 patients who demonstrated vascular injury on angiography, distal embolization was the most common choice (*n* = 84, 41.6%) with an 88.1% success rate, followed by proximal embolization (*n* = 64, 31.7%) with a 93.8% success rate and combined embolization (*n* = 54, 26.7%) with a 98.1% success rate. Apart from the rebleeding, other complications of SAE included infarction, cyst, splenic abscess, and contrast-induced renal insufficiency [7, 8, 11, 34]. In a meta-analysis of 23 studies by Rong et al. the overall incidence of major complications which required surgical intervention (CDC III) was 6.4% [11]. Reportedly, splenic abscess formation after SAE occurs in 3.8–7% of patients [8, 11, 34], and there are no current antibiotic therapy guidelines for SAE in the setting of splenic injury. At our institution, antibiotic therapy was not routinely given after SAE. Of the 202 patients who demonstrated vascular injury on angiography, six patients (3.0%) developed splenic abscess after SAE; five of these underwent CT-guided drainage, and the other, surgical drainage (Table 5). Splenic infarction was the most common post-embolization complication; however, the vast majority of these patients are asymptomatic and can be managed nonoperatively [7, 8, 11]. In our protocol, an abdominal CT scan was not routinely performed after SAE, except in selected cases; data on asymptomatic splenic infarcts are therefore lacking.

This study had several limitations. First, the retrospective nature of the study meant that the information analyzed was limited to that which appeared in the medical records. Second, as this was a single-center study, the internal structure of the trauma and interventional radiology teams may not necessarily reflect that in other hospitals.

## Conclusions

During the 15-year study period, the number of SAEs increased significantly from 4 in 2001 to 23 in 2015; conversely, the number of surgeries declined significantly from 21 (43.8%) in 2001 to 4 (10.5%) in 2015. With the incorporation of vascular injury, the total number of grade IV and V injuries in the SAE subgroup increased from 96 (41.0%) to 210 (89.7%), the 2018 AAST-OIS facilitates recognition of grade IV and V injuries. Our data confirms the superiority of the 2018 AAST-OIS compared with 1994 classifications and supports the role of SAE in changing the trend of management of BSI.
